# Editorial: The CB2 Cannabinoid System: A New Strategy in Neurodegenerative Disorder and Neuroinflammation

**DOI:** 10.3389/fnins.2017.00196

**Published:** 2017-04-06

**Authors:** Marialessandra Contino, Elena Capparelli, Nicola A. Colabufo, Ashley I. Bush

**Affiliations:** Dipartimento di Farmacia-Scienze del Farmaco, Università degli Studi di Bari Aldo MoroBari, Italy

**Keywords:** CB2R, CB2R ligands, microglial activation, neurodegenerative diseases, inflammation

The cannabinoid receptors subtype 2 (CB2R) are emerging as novel targets for the development of new therapeutic approaches and PET probes useful to early diagnose neuroinflammation as first step in several neurodegenerative disorders such as Alzheimer's disease (AD) and Parkinson disease (PD). Differently from the CB1 subtype (CB1R) present in the CNS where it modulates several functions (memory, cognition, emotion, and pain control), CB2R is mainly localized in the immune system (macrophages) and are overexpressed in microglia in response to neuroinflammation. Two microglial activation states, M1 and M2, are widely reported. The M1 state (neurotoxic state) is characterized by the release of pro-inflammatory factors (such as IL-1B, IL-18, and IL-6 and inducible nitric oxide synthase) while the M2 state (neuroprotective state) is associated to the release of anti-inflammatory factors (IL-10, IL-4, and NGF). CB2R plays a pivotal role in microglial-derived neuroinflammation, since modulates cell proliferation, migration, and differentiation into M1 or M2 phenotypes (Mecha et al., 2015).

This Research Topic is mainly focused on the involvment of CB2R in neurodegenerative disorders and on the usefulness of CB2R ligands in the therapy and early diagnosis of neuroinflammation as onset of neurodegeneration.

In the reviews of Aso and Ferrer and Cassano et al. an interesting and exaustive overview of the endogenous cannabinoid signaling and its role in neuroinflammation and neurogenesis is reported. The potential of CB2R as therapeutic target in AD is argued by several evidences derived by robust experimental models and the effects modulated by CB2R agonists on different pathways involved in the pathogenesis of AD are discussed; indeed, these ligands are able to reduce inflammation, Aβ production and deposition, tau protein hyper-phosphorylation and oxidative stress damage caused by Aβ peptides. CB2R agonists are also able to induce Aβ clearance leading to cognitive improvement in AD models.

Javed et al. focused their study on the neuroprotective effect of β-caryophyllene (BCP), a natural CB2R agonist, in a rotenone (ROT)-induced animal model of PD. In their paper, the authors reported the beneficial neuroprotective effect due to BCP activity against ROT-induced neurodegeneration; the observed beneficial effects has been linked to CB2R activation since the administration of the CB2R antagonist AM630 was able to decrease the neuroprotective effects of BCP.

Navarro-Dorado et al. in their research article deepened the beneficial effects of CBR ligands in AD-type pathology studying the effects of the CB1/CB2 mixed agonist WIN 55,212-2 and the CB2 selective agonist JWH-133 on the vessel density in amyloid precursor protein (APP) transgenic mice, line 2,576, observing an improvment of the vascular responses.

Navarro et al. considered the localization of the CB2R subtype in specific cell types (activated astrocytes, reactive microglia, perivascular microglia, oligodendrocytes, and neural progenitor cells) and structures (blood-brain barrier) pivotal for the maintenance of the CNS integrity; moreover, they underlined the challenges faced in the CB2R-based drug design for the development of CB2R PET probes and for an innovative therapeutic strategy for neurodegeneration. Therefore, the authors reported an overview of the CB2R ligands planned as therapeutic and diagnostic agents in CNS diseases. In the diagnostic field, starting from the first PET radiotracer developed to image CB2R aspect, [11C]-NE40, the structural optimization performed with the aim to improve the brain penetration and to decrease the non-specific binding of these CB2R probes has been reported. The authors identified a 18F-triazine scaffold as a promising PET tracer candidate for the *in vivo* evaluation of inflammation. In the therapeutic field, the authors showed the findings reported by CB2R agonists in *in vitro* (BV-2 microglial cell line and in primary culture of microglia) and *in vivo* models (murine model) of neurodegeneration establishing a direct link between CB2R-targeting and neuroprotecion. Despite the increasing number of CB2R ligands developed, few synthetic CB2R agonists have reached clinical trials; the CB2R agonists GW842166X, CP55940, S-777469, and JTE-907 completed phase II for the pain therapy but none of them has been used in neurodegenerative diseases. However, the *in vitro* and *in vivo* studies performed led to several scaffolds useful to the development of CB2R ligands able to exert neuroprotection and neurorestoration.

Bu et al. shed light on a new scenario in neurodegenerative diseases since suggests a new therapeutic approach by the use of CB2R inverse agonists. Indeed, in their research article the authors deepened their previous work focused on the pivotal role of the CB2R inverse agonist, SMM-189, on neuroprotection, demonstrating the ability of SMM-189 to act on brain microglia converting it to the beneficial M2 state leading to neurons rescue in cortex, striatum, and amygdala. Therefore, not only CB2R agonists but also CB2R inverse agonists could be an useful strategy to face neuroinflammation in neurodegeneration; the difference between the two CB2R profiles (agonist and inverse agonist) is that the treatment with a CB2R agonist decreases M1 activation but do not induce M2 activation state.

Ahamed et al. and Haider et al. reported the development of PET-imaging agents to target CB2R: [11C]MA2 and [18F]MA3 and [11C]AAT-015 and [11C]AAT-778.

The first two probes are 11C- and 18F- radiolabelled analogs of the highly potent arylamide oxadiazole CB2 agonist. Both the analogs displayed a good affinity toward the desired target (CB2R), good radiochemical yield, high radiochemical purity, and high specific activity. Both probes displayed good results *in vivo* since displayed an efficient blood clearance and high brain uptake. By contrast, the thiophene-based radiotracers [11C]AAT-778 and [11 C]AAT-015 developed by Haider et al. failed *in vivo* because of the lack of specificity for CB2-R positive spleen tissue and thus the two tracers were not further evaluated in neuroinflammatory animal models. However, the high CB2R affinity and selectivity makes this class of compounds the starting point for structural optimization to improve physicochemical and pharmacological properties.

In conclusion, considering that neuroinflammation has been widely reported as indicator and modulator of neurodegeneration (Wyss-Coray and Mucke, 2002), the reduction of the neuroinflammatory responses could be considered as a new therapeutic strategy in these diseases. Moreover, the selective CB2R overexpression on the activated-microglial cells provides also a highly specialized target useful to an early diagnosis of the neurodegenerative diseases.

## Author contributions

MC managed the topic and wrote the manuscript. EC, NC, and AB helped to the manage step of the topic and to the realization of the Editorial.

### Conflict of interest statement

The authors declare that the research was conducted in the absence of any commercial or financial relationships that could be construed as a potential conflict of interest.
